# A Comparative Analysis of Low and High SiC Volume Fraction Additively Manufactured SiC/Ti6Al4V(ELI) Composites Based on the Best Process Parameters of Laser Power, Scanning Speed and Hatch Distance

**DOI:** 10.3390/ma17112606

**Published:** 2024-05-28

**Authors:** Masenate Thamae, Maina Maringa, Willie du Preez

**Affiliations:** 1 Department of Mechanical and Mechatronics Engineering, Faculty of Engineering, Built Environment and Information Technology, Central University of Technology, Bloemfontein 9301, Free State, South Africa; mmaringa@cut.ac.za; 2 Centre for Rapid Prototyping and Manufacturing, Faculty of Engineering, Built Environment and Information Technology, Central University of Technology, Bloemfontein 9301, Free State, South Africa; wdupreez@cut.ac.za

**Keywords:** hatch distance, overlap rate, single layers, mechanical properties

## Abstract

Silicon carbide (SiC) exhibits intriguing thermo-physical properties such as higher heat capacity and conductivity, as well as a lower density than Ti6Al4V(ELI). These properties make SiC a good candidate for the reinforcement of Ti6Al4V(ELI) with respect to its use as a heat shield in aero turbines to increase their efficiency. The traditional materials used in aircraft structures were required to have a combination of good mechanical properties such as strength, stiffness, and hardness and low weight, as well as low thermo-physical properties such as coefficient of thermal expansion (CTE) and thermal conductivity. The alloy Ti6Al4V(ELI) has a density of 4.45 g/cm^3^, which is lower than that of structural steel (7.4 g/cm^3^) and higher than that of aluminium (2.5 g/cm^3^). Lower density benefits light weighting. Aluminium is the lightest of the traditional materials used but has relatively low strength. The CTE of SiC of 4.6 × 10^−6^/K is lower than that of Ti6Al4V(ELI) of 8.6 × 10^−6^/K, while the density of SiC of 3.21 g/cm^3^ is lower than that of Ti6Al4V(ELI) of 4.45 g/cm^3^. Therefore, from the theory of composites, SiC/Ti6Al4V(ELI) composites are expected to have lower densities and CTEs than those of Ti6Al4V(ELI), thus providing for lightweighting and less thermal related buckling or separation at their joints with carbon/epoxy resin panels. The specific strength, stiffness, and Knoop hardness of SiC of 75–490 kNm/kg, 132 MNm/kg, and 600–3800 GPa, respectively, are generally larger than those of Ti6Al4V(ELI) of 211 KNm/kg, 24 MNm/kg, and 880 GPa, respectively. Therefore, investigating reinforcement of Ti6Al4V(ELI) with SiC particles is worthwhile as it will lead to the formation of composites that are stronger, stiffer, harder, and lighter, with lower values of CTE. For additive manufacturing, this requires initial studies to optimise the process parameters of laser power and scanning speed for single tracks. To print single tracks in the present work, different laser powers ranging from 100 W to 350 W and scanning speeds ranging from 0.3 m/s to 2.7 m/s were used for different SiC volume fraction values of values. To print single layers, different values of hatch distance were used together with the best values of laser power and scanning speed determined elsewhere by the authors for different volume fractions of SiC. Through optical microscopy, the built tracks and their cross sections were examined. By using laser power and scanning speeds of 200 W and 1.2 m/s, and 150 W and 0.8 m/s, respectively, the best tracks at 5% and 10% volume fractions were obtained, whereas the best tracks at 25% volume fraction were achieved using a laser power of 200 W and a scanning speed of 0.5 m/s. Furthermore, the results showed that the maximum SiC volume percentage of 30% resulted in limited or no penetration. Therefore, it is concluded from the study that parts with improved mechanical properties can be produced at SiC volume fractions ranging from 5% to 25%, while parts produced at the high volume fraction of 30% would have unacceptable mechanical qualities for the final part.

## 1. Introduction

A composite is defined as a combination of two materials that produces better qualities than each of the individual components [1,2]. Metal matrix composites (MMCs) are made up of a metal matrix phase and a non-metallic reinforcing phase. Because of their superior strength-to-weight ratio and better properties such as ductility at high temperatures, metal matrix composites have been widely used in the aerospace and automotive industries [2,3,4]. In this study, the matrix phase is Ti6Al4V(ELI), and the reinforcement phase is SiC particles. The silicon carbide particles are randomly dispersed in a Ti6Al4V(ELI) matrix to create a composite. The SiC particles are ceramics and are stiffer and more brittle than Ti6Al4V(ELI) which is a ductile material [3,4,5,6].

Titanium is widely used in the aerospace, ordinance, and automobile industries and in biomedical applications due to its outstanding properties of high corrosion resistance, high hardness, high strength-to-weight ratio, and good stiffness-to-weight ratio [7,8,9,10,11]. Although titanium alloys have good strength, stiffness, and hardness, these properties can be further improved by using ceramic materials, which have higher mechanical properties of strength, stiffness, and hardness [11,12,13]. Silicon carbide has more intriguing thermo-physical features than Ti6Al4V(ELI), such as better heat capacity (750 J/kg·K compared to 5226.3 J/kg·K) and conductivity (120 W/m·K compared to 6.7 W/m·K), as well as a lower density (3.1 g/cm^3^ compared to 4.5 g/cm^3^ [13,14,15]. As a result, the addition of SiC particles to a Ti6Al4V(ELI) matrix results in composites with better mechanical properties in terms of specific strength, specific stiffness, and hardness than the matrix alone. The low density of SiC particles will benefit light weighting in the aircraft industry [16].

The ASTM F2792-12a [17], on additive manufacturing (AM) technologies defines additive manufacturing as the “process of joining materials to make objects from three-dimensional (3D) model data, usually layer by layer, as opposed to subtractive manufacturing methodologies”. The benefits of additive manufacturing include the production of parts with complex geometries like hollow structures and undercuts that are not possible to produce with normal subtractive methods, less waste of materials, and a reduction in the number of parts for components with complex geometries [16,17,18,19,20]. Selective laser melting (SLM) is one of the most promising AM technologies for metallic materials, which involves the complete melting of powder using a laser beam in a layer-by-layer process until a full component is produced [21,22]. A complete layer consists of the arrangement of single tracks adjacent to one another [10,23]. Single tracks form the foundation in the building of parts, and it is the bonding of adjacent tracks that creates a single layer [24,25]. Multiple layers are subsequently built on top of each other to create a full part. The quality of intertrack bonds is predicated mainly on the degree of overlap of adjacent tracks, thus the need for studies to optimise hatch distances [24,25,26] Various studies have shown that good intertrack bonding occurs where the overlap rate is more than 50% [25]. The quality of interlayer bonds, on the other hand, depends on the depth of penetration and, therefore, the necessity to examine the cross-sections of built tracks and layers [8,10]. It has been demonstrated that good interlayer bonding occurs where the depth of penetration is more than one layer to ensure remelting of the previous layer, thus leading to its fusion with the current layer. This, and the absence of unmelted powder and gas pores between adjacent layers, ensure good quality of the interlayer bonds [25,26]. Hence, the properties of built 3D parts depend on the quality of the single tracks [21,27]. In the AM of MMCs, different properties of tracks and layers are generated with high and low volume fractions of reinforcing particles due to their physical and thermo-physical properties that differ from those of the matrix. As a result, it is necessary to investigate the height, width, and depth of penetration of tracks (SiC/Ti6Al4V(ELI) composite single tracks in the present case) at low and high volume fractions of the reinforcing phase, as well as the produced layers at different volume fractions. The hatch distance, which is defined as the distance from the centre of one track to another, plays a major role in ensuring sufficient bonding between adjacent tracks to achieve high-density parts [10,20,21] and should also be investigated.

Previous studies on the effect of SiC volume fractions of 0%, 4%, 8%, 12%, 16%, and 20% on SiC/Al composites done by Min [28] found that yield strength and tensile strength of the composite increased with increasing SiC volume fraction, while elongation to failure decreased with increasing SiC volume fraction. Aigbodion et al. [29] investigated the effects of SiC reinforcement on the microstructure and properties of cast Al-Si-Fe/SiC particulate composites with SiC concentrations ranging from 0% to 25%. They reported that the hardness and strength of the composites increased to a maximum at a volume fraction of 20% and decreased at the higher volume fraction of 25%. According to the results of de De Rojas Candela et al. [13] for additively manufactured SiC/Ti6Al4V composites, increasing the SiC volume fraction increased the absorption of energy because of the lower reflectivity of SiC particles compared to that of titanium powder. This and the fact that the thermal conductivity of SiC particles was higher than that of titanium powder led to an increase in the temperature of the Ti6Al4V melt pool. The study done by Shen et al. [30] showed that the addition of metallic glass particles to a Ti6Al4V matrix led to the formation of a unique nanostructured reinforcement, which resulted in the enhancement of the strength and wear resistance of the Ti6Al4V alloy while maintaining good plasticity and fracture toughness. Wang et al. [31] investigated the microstructure and properties of diamond + TiC-reinforced Ti6Al4V titanium matrix composites manufactured by directed energy deposition and reported that the microhardness and thermal conductivity of the composite increased, while wear resistance decreased.

There are different methods used to produce metal matrix composites reinforced with SiC particles, including channel angular pressing, spark plasma sintering, sintering using an induction oven, and hot extrusion [32,33]. However, the current study mainly focuses on the formation of additively manufactured selective laser-melted SiC/Ti6Al4V(ELI) composites and narrow down, in particular, on the low and high SiC volume fraction SiC/Ti6Al4V(ELI) composite single tracks and single layers based on the best process parameters of laser power, scanning speed, and hatch distance.

Elsewhere, studies have been carried out on ultra-high-temperature ceramic materials based on ZrB_2_-SiC and HfB_2_-SiC [34,35,36] for use at ultra-high temperatures and oxidising environments of oxygen, the last property of which is enhanced by the introduction of silicon carbide [36]. These studies differ from the present study in their focus on the formation and behaviour of ceramics as opposed to the formation of SiC/Ti6Al4V(ELI composites. Other studies have confirmed the formation of multiphase intermetallic composites containing TiC_x_, Ti_5_Si_3_C_x_, Ti_5_Si_3_, TiSi_2_, and SiC phases, with values of microhardness between 0.6 and 25 GPa for different phases, due to the exposure of mixtures of Ti and SiC at temperatures higher than 600 °C [13]. This phenomenon serves the useful purpose of increasing the surface wear resistance of built components [13,37,38,39].

Although there have been studies on SiC/Ti6Al4V composites, the majority of them have focused on the mechanical and physical properties of the final 3D built parts [32]. However, no research was found on individual tracks or layers. Starting studies of AM products with three-dimensional (3D) components is a poor approach because of the attendant high costs associated with material waste in the event the process parameters are not optimal. Rather, studies should first be based on the smallest representative units of additively manufactured parts, single tracks, followed by single layers. As a result, the current research sought to determine the best SiC/Ti6Al4V(ELI) composite single tracks and layers at low and high volume fractions and therefore process parameters, thus paving the way for the production of 3D parts with superior mechanical and thermo-physical properties.

## 2. Materials and Methods

Before using the Ti6Al4V(ELI) powder in AM, it was sieved with an 80 µm manual sieve to eliminate any agglomerates and undesirably large particles in order to ensure a uniform consistency of the powder. The sieved Ti6Al4V(ELI) powder was then mixed with SiC powder in a multi-tube rotary batch mixer with five tubes of 45 mm diameter and 160 mm length, each with a sealed container containing a mixture of SiC and Ti6Al4V(ELI) powders of selected volume fractions for mixing. Because there were six containers, each bearing a different volume fraction of the mixture, only four containers were used for mixing during the first round, and the last two were used in the second round of mixing. The powders were mixed for thirty minutes, and then the morphology of the mixed and different powders before mixing was determined in a JSM-6610 scanning electron microscope (SEM) supplied by JEOL, Tokyo, Japan. The Ti6Al4V(ELI), SiC, and mixed powders, the latter for different SiC volume fractions, were sampled using a spatula and mounted on a double-sided carbon tape for investigation in the SEM. The shape, size, and surface topography of the individual powders as well as the mixed powders were studied. The chemical composition of the SiC powder as determined through X-ray fluorescence analysis by Industriekeramik Hochrhein GmbH (IKH), Wutöschingen, Germany, was provided by the supplier, Weartech (Pty) Ltd., Wadeville, South Africa. The chemical composition of the Ti6Al4V(ELI) powder was determined at the Pelindaba Analytical Laboratories of the South African Nuclear Energy Corporation (NECSA), using the inductively coupled optical emission spectroscopy (ICP-OES) method. The particle size distribution (PSD) of both powders was also determined at NECSA.

The study specimens were built using a direct metal laser sintering (DMLS) EOSINT M280 machine, supplied by Electro-Optical Systems GmbH (EOS), Munich, Germany. The equipment was fitted with a 400-watt continuous-wave ytterbium-fibre laser. The laser beam had a TEM00 Gaussian shape with a spot size of 80 µm. In the building chamber, argon was employed to provide an inert protective atmosphere, and single tracks were built on a titanium substrate of size 50 × 50 mm. Single tracks were built with different levels of power of 100 W, 150 W, 200 W, 250 W, 300 W, and 350 W, as well as different scanning speeds ranging from 0.3 m/s to 2.5 m/s and SiC volume fractions of 5%, 10%, 25%, and 30%. To improve the statistical relevance of average track readings, three tracks were created for each scanning speed. A prior investigation using the same EOSINT M280 equipment generated values of the best process parameters of laser power and scanning speed for Ti6Al4V(ELI) of 200 W and 1.2 m/s [40]. These values were used as starting points in the present study, and the laser power and scanning speed then varied three steps below and above these best values, as shown in Table 1.

According to the literature, the best hatch distance experimentally determined for Ti6Al4V was 80 µm, with track widths ranging from 80 µm to 160 µm [25]. Therefore, this was used as a starting point to determine the best hatch distances for the different SiC/Ti6Al4V(ELI) composites. The hatch distances were adjusted to generate three values below and three values above the best hatch distance of 80 µm. The hatch distances used to build SiC/Ti6Al4V(ELI) single layers were 50 µm, 60 µm, 70 µm, 80 µm, 90 µm, 100 µm, and 110 µm, and these values of hatch distances were kept constant for all the volume fractions of (5%, 10%, 15%, 20%, 25%, and 30%). Multiple tracks were then built for each hatch distance to form single layers. At each volume fraction of SiC, the laser power and laser scanning speed parameter sets were kept constant at the best values determined in the testing and analysis of single tracks in earlier work by the authors [8] for different values of hatch distance.

A Zeiss Axio Scope.A1 optical microscope supplied by Carl Zeiss AG, Oberkochen, Germany was used for the assessment of the top surface images of the manufactured tracks to evaluate surface roughness, continuity of tracks, necking, and balling. The optimum tracks were selected, and their process parameters were further used in the building of single layers at different hatch distances of 50 µm, 60 µm, 70 µm, 80 µm, 90 µm, 100 µm and 110 µm. Subsequently, electrical discharge wire cutting was performed perpendicular to the length of the built tracks and single layers to obtain cross-sections of the single tracks and layers for analysis. These obtained cross-sections were then mounted in resin using a Struers Cito Press-1 machine and polished on a Struers Tegramin-25 polishing machine (Struers, Cleveland, OH, USA) before being examined in the JSM-6610 SEM. The geometrical characteristics of width, depth, and height of the tracks were all measured, and keyhole and undercutting features were explored. In addition, the overlap rate and bonding of adjacent tracks in single layers were analysed on the cross-sections for each SiC volume fraction in a SiC/Ti6Al4V(ELI) composite.

The critical process parameters of laser power, scanning speed, and hatch distance were varied in this study, in order to select the best ones that produced single tracks and layers with the best top surfaces and cross-sectional morphologies for further use in building 3D parts. The best top surfaces were those with more even widths, no pre-balling or balling, no keyholes, and little or no spatter particles and unmelted powder [8,32], The best cross-sections were those that elicited the optimum depth-to-width ratio of 0.5, had wide semi-circular top track cross-sections, modest track heights, no keyholes, no balling, no humps or undercutting, and minimal porosity [8]. However, the layer thickness and the beam diameter were kept constant during the process.

## 3. Results and Discussion

Here, in four subsections, the results obtained for different sets of process parameters and SiC volume fractions in single tracks and single layers are presented and discussed.

### 3.1. Particle Size Distribution and Morphology of SiC and Ti6Al4V(ELI) Powders

**Figure 1:**
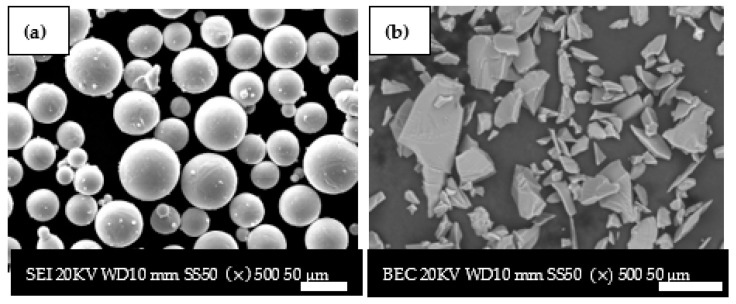
SEM images of powders: (**a**) SE image of Ti6Al4V(ELI) particles and (**b**) BE image of SiC particles.

The overall shape of the Ti6Al4V (ELI) particles was confirmed to be spherical with a particle size range of 20–50 µm, while the SiC particles were confirmed to be irregular with a particle size range less than 45 µm, as evident in the SEM micrographs shown in Figure 1. These results for spherical powder were also reported by Brick et al. [41]. They further reported that powder with high sphericity leads to good packing density and flowability, thus enabling the fabrication of parts with high density and good mechanical properties.

The chemical composition of SiC and Ti6Al4V(EL) powders that were provided by the suppliers, Weartech (Pty) Ltd., Wadeville, South Africa, and TLS Technik GmbH (Bitterfeld-Wolfen, Germany), respectively, is presented in Table 2.

Table 2 shows that the supplied SiC powder had very small amounts of trace elements with a percentage less than 0.1, while over 99% of it was pure SiC powder. The Ti6Al4V(ELI) powder, on the other hand, was composed of 89.56% titanium powder alloyed with Aluminium (6%) and vanadium (4%), with some small trace elements of iron, oxygen, nitrogen and hydrogen.

**Figure 2:**
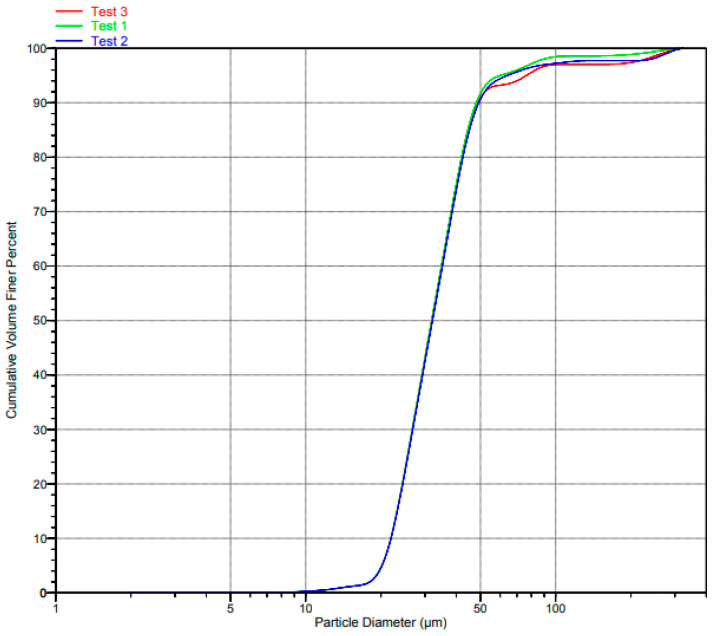
PSD diagram of the Ti6Al4V(ELI) particles.

**Figure 3:**
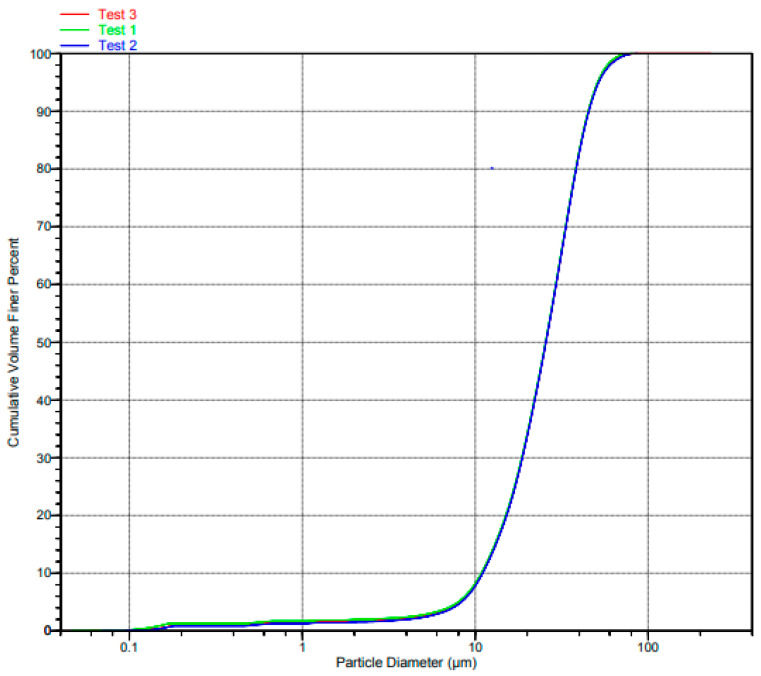
PSD diagram of the SiC particles.

The PSD of Ti6Al4V(ELI) and SiC are shown in Figure 2 and Figure 3, respectively.

It is evident from the graphs in the foregoing two figures that the test data 1, 2, and 3 are indistinguishable over most of the central range of the volume cumulative curves, barely distinguishable at the two extreme ends, and significantly distinguishable at the upper extreme end for the curves of Ti6Al4V(ELI).

### 3.2. SEM Micrographs of the Mixed SiC and Ti6Al4V(ELI) Powders

**Figure 4:**
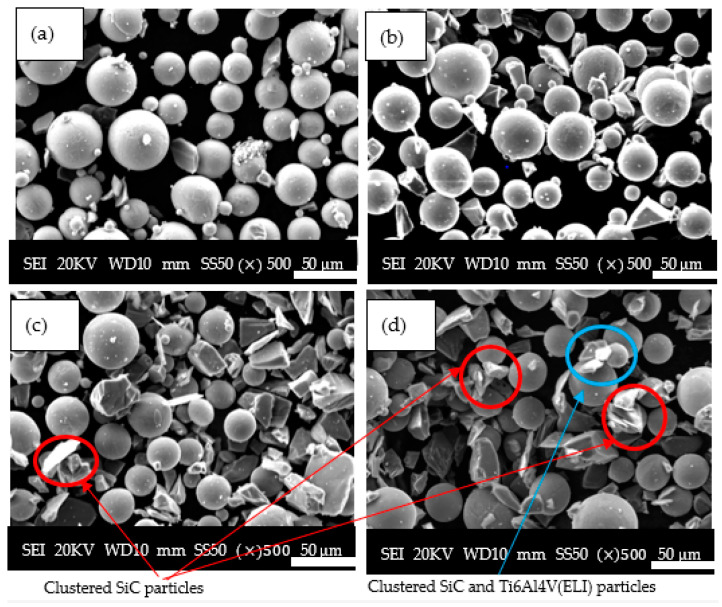
SEM SE micrographs of SiC/Ti6Al4V (ELI) mixtures at different SiC volume fractions, (**a**) 5% SiC vol. fraction, (**b**) 10% SiC vol. fraction, (**c**) 25% vol. fraction, and (**d**) 30% vol. fraction.

The results of the SEM SE micrographs of SiC/Ti6Al4V(ELI) mixtures at low volume fractions of 5% and 10% as well as high volume fractions of 25% and 30% are shown in Figure 4.

Figure 4 shows that, for 5–10% volume fractions, the SiC particles were dispersed randomly among the Ti6Al4V(ELI) particles without clustering. Clustering of particles was seen to occur at 25% and 30% SiC volume fractions. The clustering of SiC particles is indicated by the red circles, while that of Ti6Al4V (ELI) with SiC particles is indicated by the blue circles in the micrographs presented in this figure. Therefore, the agglomeration of these particles increases with the loading of SiC particles. The agglomeration of powder at high SiC volume fractions is expected to cause the formation of irregularities during the deposition of powder layers, which, according to the findings of Liets et al. [42], influences the width of the molten track during sintering.

### 3.3. Top Surface and Cross-Sectional Analysis of Single Tracks at 5% and 10% SiC Volume Fraction in SiC/Ti6Al4V(ELI) Composites

**Figure 5:**
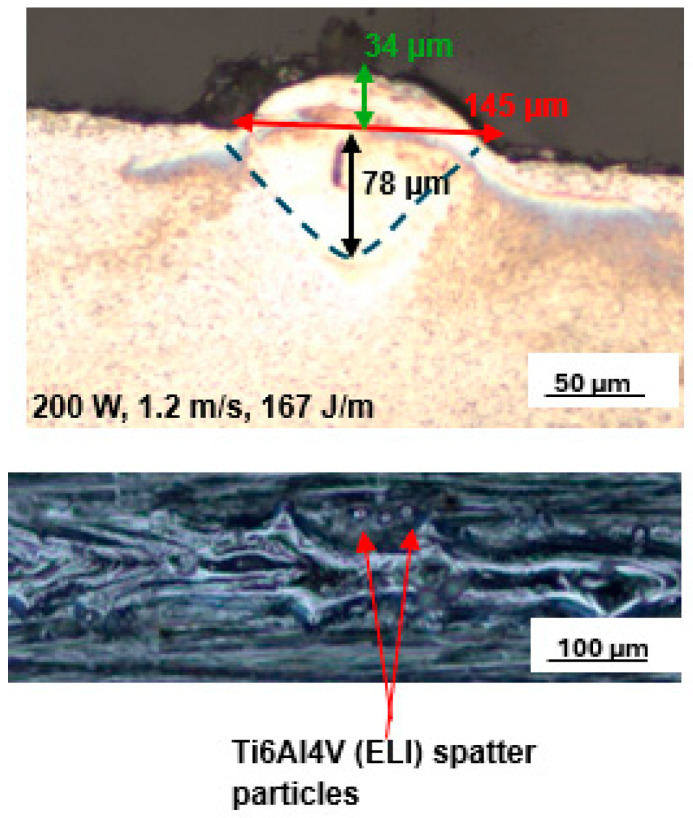
Cross-section and top surface topography of the best single track at 5% SiC volume fraction in an SiC/Ti6Al4V(ELI) composite built at 200 W, 1.2 m/s, and 167 J/m.

**Figure 6:**
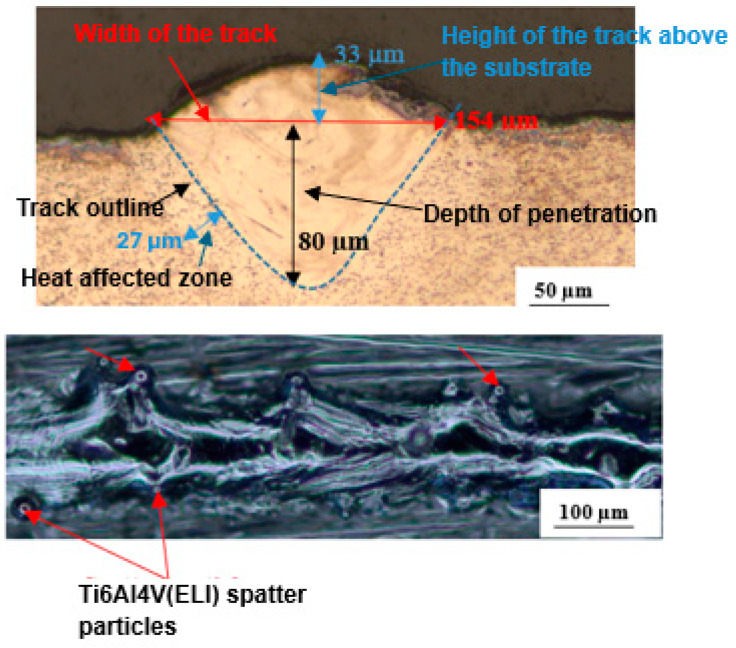
Cross-section and top surface topography of the best single track at 10% SiC volume fraction in an SiC/Ti6Al4V(ELI) composite built at 150 W, 0.8 m/s, and 188 J/m.

Numerous single tracks were built and analysed at each of the selected SiC volume fractions for the laser scanning speeds of 0.3 m/s to 0.9 m/s at different values of power from 100 W to 350 W. The best single tracks at 5% and 10% SiC volume fractions in a SiC/Ti6Al4V(ELI) composite were obtained at the process parameters of laser power, scanning speed, and linear energy density of 200 W, 1.2 m/s, and 167 J/m, and 150 W, 0.8 m/s, and 188 J/m, respectively. The top surface scans and cross-sections of the best tracks at 5% SiC volume fraction are shown in Figure 5, while those at 10% SiC are shown in Figure 6. From these images, the surface morphology and geometrical characteristics of width, depth of penetration, and height above the substrate of the tracks were determined.

The top surface image of the best single track at the lowest SiC volume fraction of 5% revealed a continuous melted track with some spatter particles at the track’s borders. However, as the SiC volume fraction increased to 10%, the number of spatter particles increased. It should be noted that the linear energy densities utilised to build the tracks at 5% and 10% SiC volume fractions differed. The track at 10% SiC volume fraction was built at a linear energy density of 188 J/m, while the track at 5% SiC volume fraction was built at a lower linear energy density of 167 J/m, which was similar to the best process parameters of Ti6Al4V.

The presence of spatter particles may be because when the laser moves across the powder bed, it does not only melt the powder particles beneath it, but also attempts to melt the particles at the edges of the track [26,43]. As a result of the Gaussian distribution of energy in the beam, the energy density at the track’s periphery is insufficient to entirely melt the particles, resulting in partially melted particles near the edges of the solidified track, and they thus appear there as spatter particles [44].

Additionally, the increased linear energy density at the higher volume fraction of SiC raised the temperature of the melt pool, resulting in a stronger wave current within the melt pool due to the Marangoni effect [45]. Because of the increased wave current, the molten pool pinched off powder ahead of the laser spot. Thus, the increase of spatter particles at the higher linear energy density in the current experiment is similar to the findings of reference [25,26]. The reduced laser scanning speed of 0.8 m/s at a 10% SiC volume fraction contributed to the increased linear energy density. This is consistent with the reported fact that reduced laser scanning speed results in metal powders retaining more energy [24]. Furthermore, increasing the SiC volume percentage increases the energy absorption of the composite because SiC particles have lower reflectance and a higher heat capacity than titanium powder. Similar to the findings in [11], the width of tracks increased with increasing linear energy density. According to Equation (1), linear energy density (LED) is defined as the ratio of laser power over laser scanning speed; thus, decreasing the laser scanning speed at fixed power increases the linear energy density [43,44].

(1) $$\mathrm{LED}=\frac{P}{V}\;(J/m)$$

where the symbols P and V represent laser power and laser scanning speed, respectively. At 10% SiC volume fraction, the depth of penetration is seen in the two figures to be slightly higher (80 µm) than the 78 µm depth at 5% SiC volume fraction. The height of the track dropped slightly from 34 µm at 5% SiC volume fraction to 33 µm at 10% SiC volume fraction. Furthermore, a 6.2% increase in the width of the track from 145 µm at 5% SiC volume fraction to 154 µm at 10% SiC volume fraction was observed. The heat-affected zone (HAZ) also increased with an increasing SiC volume fraction. This was because the higher heat conductivity induced by the higher percentage of SiC particles led to an increased transfer of heat to the surrounding particles, thus resulting in an increased HAZ.

From the foregoing examination of the top surface and cross-sections of single tracks at 5% and 10% SiC volume fractions, it is clear that there was a relatively small difference in the geometrical properties of the depth and height of the tracks at these low volume fractions.

### 3.4. Top Surface and Cross-Sectional Analysis of SiC/Ti6Al4V(ELI) Single Layers at 5% and 10% SiC Volume Fractions in SiC/Ti6Al4V(ELI) Composites

**Figure 7:**
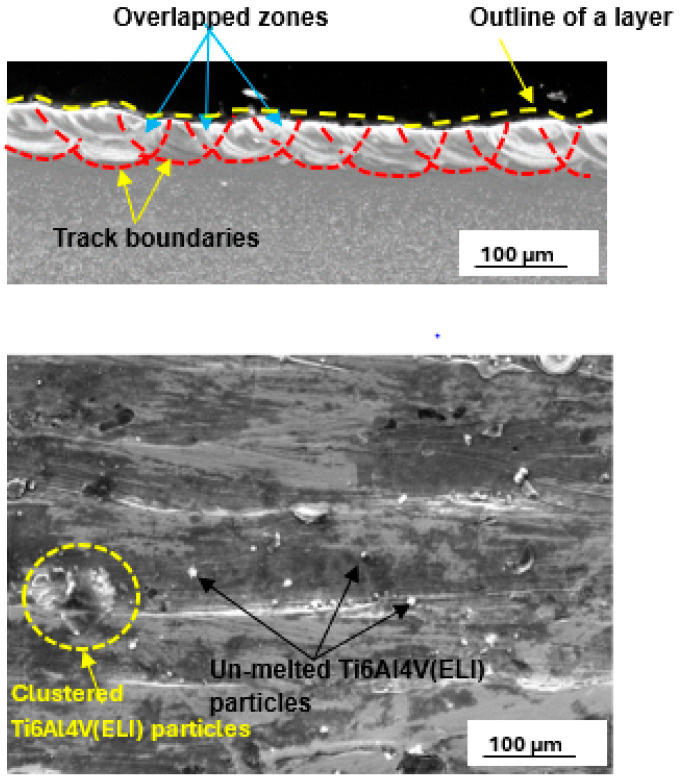
Cross-section and top surface view of the best single layer at 5% SiC volume fraction in an SiC/Ti6Al4V(ELI) composite, produced at a hatch distance of 60 µm.

The best parameter sets for single tracks produced at 5% and 10% SiC volume fractions were further used to build single layers. A single layer consists of arrangements of single tracks whose centres are separated by a hatch distance. In this study, the hatch distances used were 50 µm, 60 µm, 70 µm, 80 µm, 90 µm, 100 µm, and 110 µm. From the single layers built at these hatch distances, the hatch distance of 60 µm was identified as producing the best surface layer for 5% SiC, and this layer is presented in Figure 7.

**Figure 8:**
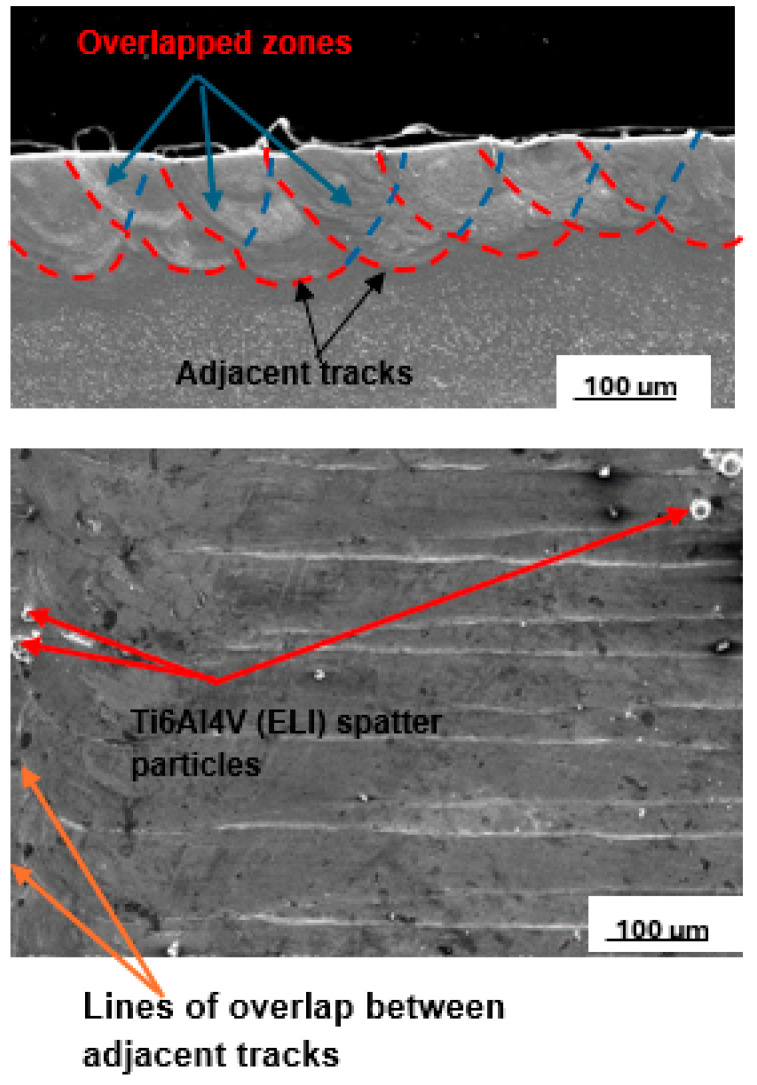
Cross-section and top surface view of the best single layer at 10% SiC volume fraction in an SiC/Ti6Al4V(ELI) composite, printed at a hatch distance of 70 µm.

Figure 8 shows the best layer obtained at a 10% SiC volume fraction, from which the depth of penetration and overlap rate were determined for the optimal hatch distance of 70 µm.

The degree of overlap of the tracks was determined from the overlap rate, which plays a key role in influencing the mechanical properties of the built part and is calculated using Equation (2) [44,46].

(2) $$\varphi =1-\frac{h}{W}$$

where the variables φ, h, and W, represent the overlap rate, hatch distance, and average track width, respectively.

From the results obtained for the different hatch distances, the most even and smooth layer at 5% SiC volume fraction was obtained at a hatch distance of 60 µm, with an overlap rate of 67%. This overlap rate is high enough to re-melt adjacent tracks, resulting in effective metallurgically bonded tracks with no inter-track porosity. This observation is consistent with the findings of Ramosena et al. [47] and Guan et al. [48], who found that sufficient overlap between tracks resulted in no gaps and the delamination of tracks in a layer. Dong et al. [44] also found that a suitable overlap rate is essential for efficient densification of fabricated samples, resulting in little to no porosity between single tracks.

The yellow dotted line on the cross-section in Figure 3 demonstrates the difference in height of adjacent tracks, which can be further enhanced by utilising a 50% offset scanning strategy. Height differences between adjacent tracks result in the non-uniform deposition of ensuing layers of powder, as found by Ren et al. [46]. Smoother layers with fewer surface flaws have been documented in the literature to result in reduced relative density of produced parts, which improves mechanical characteristics [43,44]. The top view of the layer in Figure 3 shows continuous melted tracks with some clustered and unmelted Ti6Al4V(ELI) particles. These clustered and unmelted particles will contribute to the non-uniformity of the subsequent layer. Application of a rescanning strategy to the surface of such a layer will result in a smoother surface [25,48].

For the 10% SiC volume fraction, the best layer was obtained at 70 µm hatch distance and an overlap rate of 59%, with the parameter sets of laser power, scanning speed, and linear energy density of 200 W, 1 m/s, and 200 J/m, respectively. The top view of this layer, presented in Figure 4, shows continuous tracks that are relatively smooth and with fewer Ti6Al4V(ELI) spatter particles than in the previous case. Such a relatively smooth layer will lead to a fairly uniform deposition of the next layer of powder during the building of 3-D parts. Ren et al. [46] observed that obtaining continuous single tracks well bonded to the tracks in previous layers was a requirement for producing parts with high densities.

Clearly, the selected parameter sets for single tracks at 5% and 10% SiC volume fractions, with the best hatch distances employed in building single layers, resulted in sufficient track overlap, indicating that these process parameters of laser power, scanning speed, linear energy density, and hatch distance, can be used to build good-quality 3D parts.

### 3.5. Top Surface and Cross-Sectional Analysis of SiC/Ti6Al4V(ELI) Single Tracks at 25% and 30% SiC Volume Fractions in SiC/Ti6Al4V(ELI) Composites

**Figure 9:**
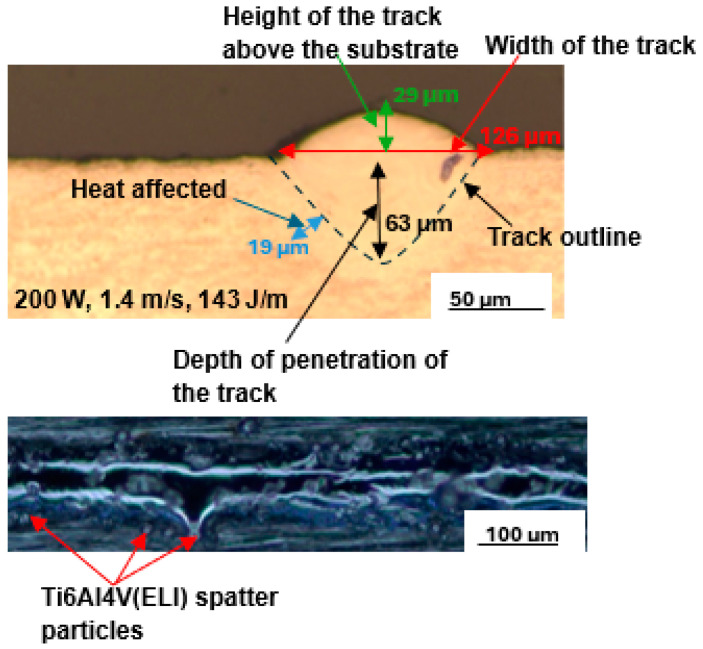
Cross-section and top surface view of the best single track at 25% SiC volume fraction in an SiC/Ti6Al4V(ELI) composite built at 200 W, 1.4 m/s, and 143 J/m.

An example of the best single tracks produced at a 25% SiC volume fraction built with the process parameters of laser power, scanning speed, and energy density of 200 W, 1.4 m/s, and 143 J/m is presented in Figure 9.

The track’s top surface view in Figure 5 shows a continuous melted track with a few spatter particles at the edges. The U-shape profile in the cross-sectional view in Figure 5 indicates that the half-width of the track is equal to the depth of penetration [32,47]. These findings are consistent with Eager and Tsai’s [33] model, who found that the melting conduction mode was semi-circular. This implied that the optimal process parameter set would produce a track with a depth-to-width ratio of 0.5. If the ratio is less than 0.5, it implies poor conduction melting with a shallow depth of penetration, whereas a number greater than 0.5 suggests the keyhole effect with a V-shape profile and a depth of penetration greater than half the track width [26,49].

**Figure 10:**
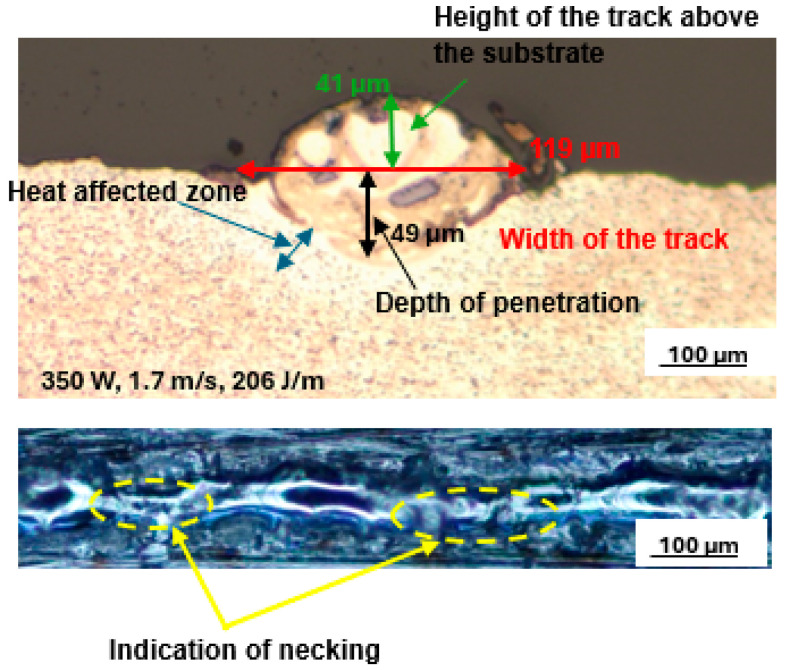
Cross-section and top surface view of the best single track at 30% SiC volume fraction in an SiC/Ti6Al4V(ELI) composite produced at 350 W, 1.7 m/s, and 206 J/m.

The top surface view of the track at the highest SiC volume fraction of 30% created at 350 W, 1.7 m/s, and 206 J/m is shown in Figure 10.

The top surface scan of the track in Figure 6 showed a narrow track with fewer spatter particles and signs of necking. This was due to the increased surface tension and viscosity of the melt, which increased with decreasing temperature. Further examination of the cross-section in Figure 6 showed a width of 119 µm and a depth of penetration of 49 µm, which corresponds to a depth-to-width ratio of 0.4. This ratio is less than the optimum value of 0.5, which is an indication of low depth of penetration. Dzogbewu et al. [25] reported that low depth of penetration occurred due to insufficient energy density to melt and penetrate the substrate. Low depth of penetration results in insufficient bonding of layers with the substrate or the previous layers [47]. Therefore, this set of process parameters is not suitable for the building of 3D parts and is likely to lead to the formation of defects during multilayer manufacturing processes [47,48,50,51].

When comparing the cross-sections of the tracks at 25% and 30% SiC volume fractions, a decrease of 5.5% and 22% in the width of the track and depth of penetration, respectively, from values of 126 µm and 63 µm for the 25% volume fraction to 119 µm and 49 µm for the 30% volume fraction were found. Additionally, an increase of 40% in the height of the track was observed with a rising SiC volume fraction from 25% to 30%. Considering the influence of the thermal properties of the SiC particles, which include high heat capacity and conductivity, it was expected that, for higher volume fractions of SiC, higher linear energy densities would be required to obtain a temperature of the melt that would be sufficient for optimal depth of penetration.

The results in Figure 6 were achieved at a higher linear energy density of 206 J/m than that for the results in Figure 5. Adequate penetration into the substrate and width of tracks were thus expected in the former case. However, the cross-sections obtained at the higher linear energy density of 206 J/m showed that the laser beam still did not penetrate the substrate adequately and led to tracks with lower widths and higher heights, all signs of inadequate heat. These results confirmed that the LPBF melting process is non-linear and that optimum parameters can only be attained through careful evaluation and pairing of the laser power and laser scanning speed. Similar results of non-linearity in the LPBF were reported by [26]. Another possibility is that SiC particles floated on top of the melt pool and absorbed a substantial amount of energy without fully transferring it to Ti6Al4V particles because of their physical and thermal features, which include low density, high heat capacity, and poor reflectance. As a result, the Ti6Al4V(ELI) received less energy. Furthermore, due to the relatively low conductivity of the Ti6Al4V(ELI) particles, they did not get enough heat to completely melt the powder and penetrate the substrate.

### 3.6. Top Surface and Cross-Sectional Analysis of SiC/Ti6Al4V(ELI) Single Layers at 25% and 30% SiC Volume Fractions in SiC/Ti6Al4V(ELI) Composites

**Figure 11:**
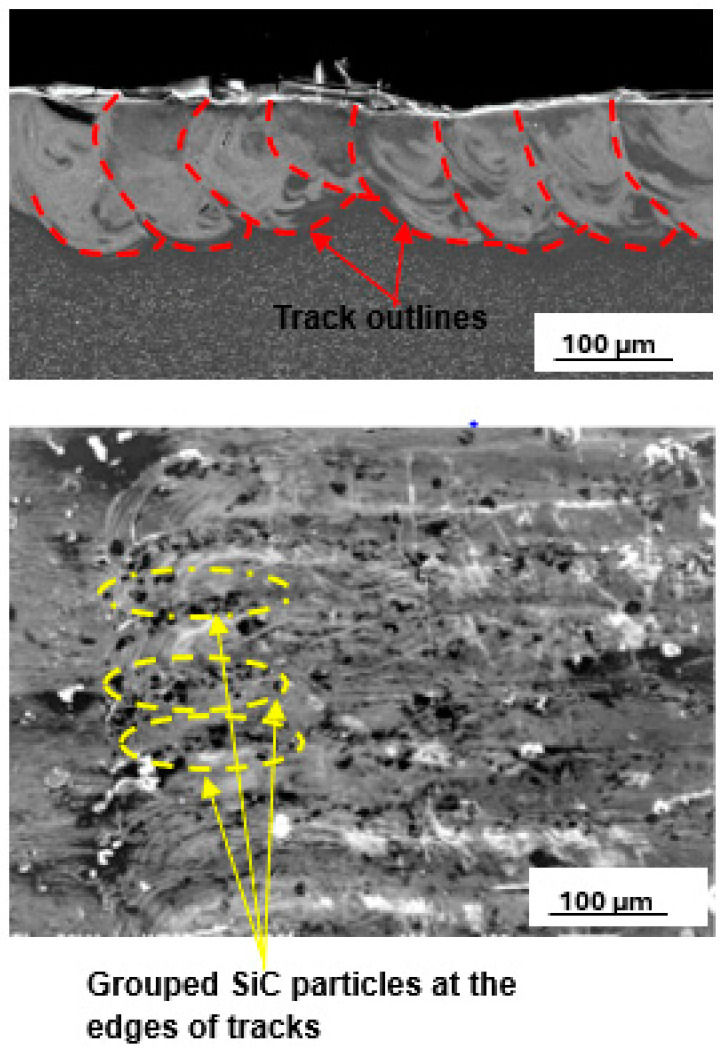
Cross-section and top surface view of the best single layer at 25% SiC volume fraction in an SiC/Ti6Al4V(ELI) composite, produced at the best hatch distance of 60 µm.

The best layer at 25% SiC volume fraction, which is shown in Figure 11, was obtained at a hatch distance of 60 µm with process parameters of laser power, scanning speed, and linear energy density of 200 W, 1.2 m/s, and 167 J/m, respectively.

The hatch distance of 60 µm produced a layer with an overlap rate of 59%. An overlap of 50% and above has been reported to provide good bonding between the tracks [44]. Moreover, considering the smooth top surface of the upper image shown in Figure 7, it is expected that smooth layers with continuous tracks would result in the production of layers with good interlayer bonding [48,49]. The clusters of SiC particles observed at the edges of the tracks in the lower image in Figure 7 are attributed to the low density of the SiC particles, leading to their being pushed by the Marangoni forces to the edges of the melt [23].

**Figure 12:**
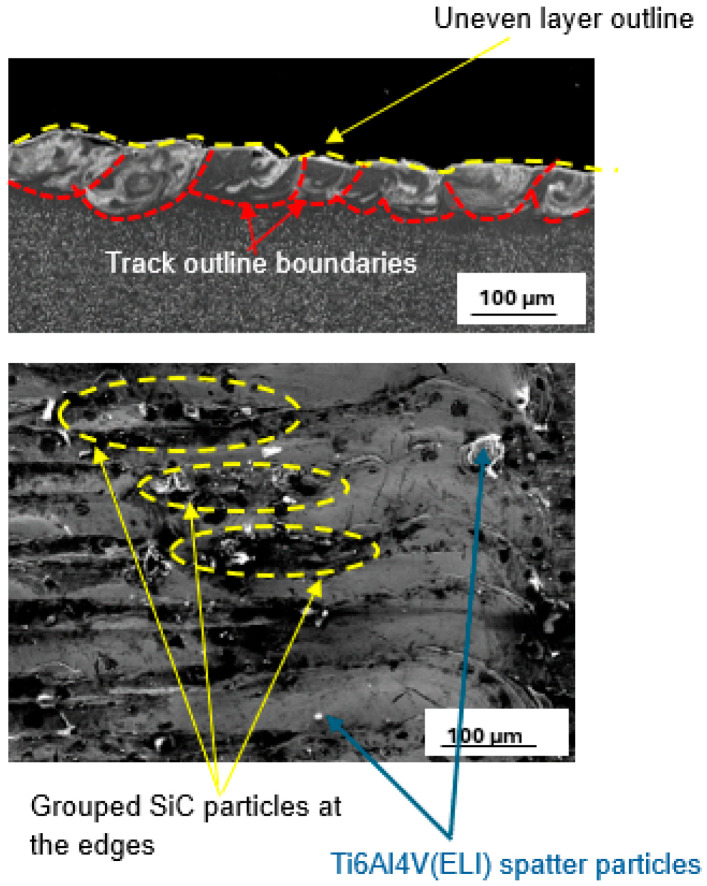
Cross-section and top surface view of the best single layer at 30% SiC volume fraction in SiC/Ti6Al4V(ELI) composite, produced at the best hatch distance of 50 µm.

Figure 12 shows a layer produced with the best set of process parameters at the highest SiC volume fraction of 30%. The best hatch distance at 30% SiC volume fraction in the SiC/Ti6Al4V(ELI) composite was observed to be 50 µm. The printed layer was noted to have clusters of SiC particles at the edges of the tracks and surface irregularities. These surface features will have a negative impact on the microstructural and mechanical properties of the built parts [44,50].

The cross-section shown in Figure 8, had an overlap rate of 64%, indicating adequate inter-track bonding and hence suitability for creating 3D parts. At this high SiC volume fraction, however, the layer penetration is suboptimal. Such shallow penetration would not offer sufficient bonding with the prior layer, resulting in delamination [49,51,52,53]. Therefore, at this volume fraction of SiC, the process parameters used are not suited for building 3D-built parts.

## 4. Comparative Analysis of Geometrical Characteristics of Low and High SiC Volume Fractions

**Figure 13:**
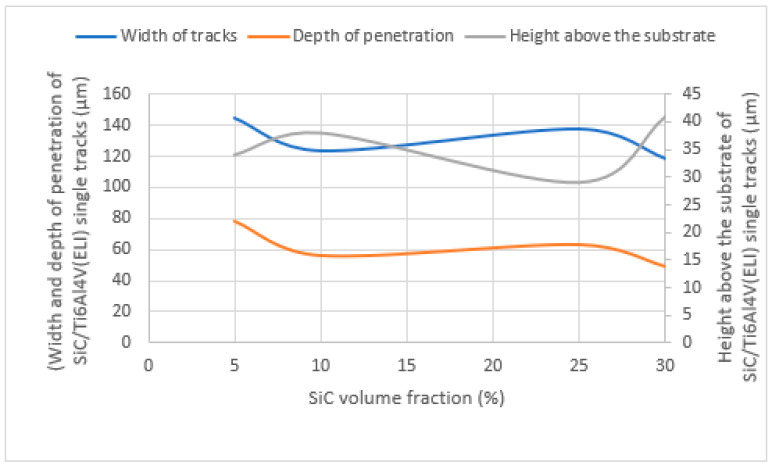
Graphs of width, height above the substrate, and depth of penetration of SiC/Ti6Al4V(ELI) single tracks built at high and low volume fractions of SiC.

From the analysis of the best cross-sections of SiC/Ti6Al4V(ELI) single tracks discussed in this paper, a summary of the geometrical characteristics of width of tracks, depths, and heights of tracks above the substrate of the best single tracks at high and low SiC volume fractions produced from SiC volume fractions of 5%, 10%, 25%, and 30% is represented by the graphs in Figure 13.

As seen in Figure 13, the width of built tracks and the depth of penetration of SiC/Ti6Al4V(ELI) single tracks displayed a similar pattern. The highest points from both graphs were reached at 5% SiC volume fraction. A decreasing trend of both widths of built tracks and their depth of penetration was observed at low volume fractions of 5% and 10% and at high volume fractions of 25% and 30%. Nevertheless, the height of the tracks did not show the same behaviour. There was an increase from 5% to 10%, while a decreasing trend was observed between 25% and 30% SiC volume fraction. The thermo-physical properties of SiC of low density, low reflectance, high heat capacity, and high conductivity negatively affected the depth of penetration and widths of tracks at low and high SiC volume fractions, where a declining trend was observed. Because of the low density of SiC particles and the cluster of SiC powder particles at high volume fractions, it is thought that the SiC particles floated on top of the Ti6Al4V(ELI) melt at high volume fractions and acted as heat shields, limiting the transfer of laser power to fully melt Ti6Al4V(ELI) particles and penetrate the substrate, thus leading to the formation narrower tracks with smaller depths of penetration.

The present work was focused solely on single tracks and single layers, and thus, there was no case of interlayer bonding, which in the case of multiple layers would occur in the form of interlayer fusion between layers in the laser melting of Ti6Al4V (ELI). The analysis of the geometries of the cross-sections of the built tracks did provide a guide on the effectiveness of bonding expected between the layers [8]. Thus, wide tracks with semi-circular cross-sections and reasonable heights are expected to provide large surfaces for bonding and, therefore, good bonding between layers [8].

## 5. Conclusions

- Experiments performed in this study indicated the possibility of producing parts with improved mechanical properties for volume fractions of SiC in SiC/Ti6Al4V(ELI) composites ranging from 5% to 25%.
- From the qualitative analysis of surface roughness by observing clustering of SiC particles at the edges of tracks as well as surface imperfections and their variation with the volume fraction of SiC, it was confirmed in this study that the surface roughness of layers increased with rising SiC volume fractions from 25% to 30% due to clusters of SiC particles at the edges of the tracks, and at lower volume fractions, due to an increased number of spatter particles at the edges of tracks.
- The single tracks and single layers produced at the highest SiC volume fraction of 30% used in the present work had unacceptable geometrical and surface features; therefore, this indicated that the building of parts at the high volume fraction of 30% would lead to unacceptable mechanical qualities.
- Additionally, it can be concluded that the thermal properties of SiC particles have a negative effect at high volume fractions on the penetration of single tracks and single layers and will lead to a reduction in the mechanical properties of parts. It is concluded that the building of multiple layers and 3D parts can be pursued based on the optimal process parameter sets of hatch distance, laser power, and scanning speed found for single tracks and layers for volume fractions of SiC at and below 25%.

## Figures and Tables

**Table 1:** The process parameters used for building SiC/Ti6Al4V(ELI) single tracks.

100 W	Scanning speed (m/s)	0.3	0.4	0.5	0.6	0.7	0.8	0.9
	Linear energy density (J/m)	333	250	200	167	143	125	111
150 W	Scanning speed (m/s)	0.6	0.7	0.8	0.9	1	1.1	1.2
	Linear energy density (J/m)	250	214	188	167	150	136	125
200 W	Scanning speed (m/s)	0.8	0.9	1	1.2	1.4	1.5	1.6
	Linear energy density (J/m)	250	222	200	167	143	133	125
250 W	Scanning speed (m/s)	1.1	1.2	1.3	1.5	1.7	1.8	1.9
	Linear energy density (J/m)	227	208	192	167	147	139	132
300 W	Scanning speed (m/s)	1.2	1.4	1.6	1.8	2	2.2	2.4
	Linear energy density (J/m)	250	214	188	167	150	136	125
350 W	Scanning speed (m/s)	1.5	1.7	1.9	2.1	2.3	2.5	2.7
	Linear energy density (J/m)	233	206	184	167	152	140	130

The items in red in this table represent the values of the best process parameters mentioned in the paragraph above this table, with a linear energy density of 167 J/m, that were used as starting points, above and below which different values of laser scanning speed were used.

**Table 2:** The chemical composition of the Ti6Al4V (ELI) and SiC powders.

Ti6Al4V(ELI)	Elements	Ti	Al	V	Fe	O	N	H
	Composition (%)	89.56	6.35	3.73	0.17	0.13	0.05	0.012
SiC	Elements	Al	Ca	Ti	Fe	Y	SIC	
	Composition (%)	<0.1	<0.1	<0.1	<0.1	<0.1	99	

## Data Availability

Data contained within the article.
